# Biomarkers of Trastuzumab-Induced Cardiac Toxicity in HER2- Positive Breast Cancer Patient Population

**DOI:** 10.3390/cancers14143353

**Published:** 2022-07-10

**Authors:** Aleksandra Grela-Wojewoda, Mirosława Püsküllüoğlu, Beata Sas-Korczyńska, Tomasz Zemełka, Renata Pacholczak-Madej, Wojciech M. Wysocki, Tomasz Wojewoda, Agnieszka Adamczyk, Joanna Lompart, Michał Korman, Anna Mucha-Małecka, Marek Ziobro, Ewa Konduracka

**Affiliations:** 1 Department of Clinical Oncology, Maria Sklodowska-Curie National Research Institute of Oncology, Kraków Branch, Garncarska 11, 31-115 Kraków, Poland; mira.puskulluoglu@gmail.com (M.P.); tomasz.zemelka@onkologia.krakow.pl (T.Z.); renata.pacholczak@uj.edu.pl (R.P.-M.); joanna.lompart@onkologia.krakow.pl (J.L.); marek.ziobro@onkologia.krakow.pl (M.Z.); 2 Department of Oncology, Radiotherapy and Translational Medicine, Institute of Medical Sciences, University of Rzeszów, 35-959 Rzeszów, Poland; b.sas.korczynska@gmail.com; 3 Department of Anatomy, Jagiellonian University Medical College, 31-008 Kraków, Poland; 4 Faculty of Medicine and Health Sciences, Andrzej Frycz Modrzewski Krakow University, 30-705 Kraków, Poland; wwysocki@mp.pl (W.M.W.); wojtom@mp.pl (T.W.); 5 Department of General, Oncological and Vascular Surgery, 5th Military Clinical Hospital in Kraków, 30-006 Kraków, Poland; 6 The Maria Skłodowska-Curie National Research Institute of Oncology, Scientific Editorial Office, Wawelska 15/B, 02-781 Warszawa, Poland; 7 Department of Tumour Pathology, Maria Sklodowska-Curie National Research Institute of Oncology, Kraków Branch, Garncarska 11, 31-115 Kraków, Poland; agnieszka.adamczyk@onkologia.krakow.pl; 8 Faculty of Medicine, Jagiellonian University Medical College, 31-008 Kraków, Poland; michal.korman@student.uj.edu.pl; 9 Department of Radiotherapy, Maria Sklodowska-Curie National Research Institute of Oncology, Kraków Branch, Garncarska 11, 31-115 Kraków, Poland; anna.mucha-malecka@onkologia.krakow.pl; 10 Department of Coronary Disease and Heart Failure, Jagiellonian University Medical College, John Paul the Second Hospital, 31-008 Kraków, Poland; ekonduracka@interia.eu

**Keywords:** HER-2 positive breast cancer, cardiotoxicity, cardio-oncology, cardiac markers, trastuzumab

## Abstract

**Simple Summary:**

Trastuzumab administered as a (neo)adjuvant therapy in radically treated Human Epidermal Growth Factor Receptor 2 (HER2)-positive breast cancer patients improves overall survival. This study aimed to assess if factors commonly thought to play a role as biomarkers of trastuzumab-induced cardiotoxicity (TIC) are pathognomonic for this injury. Data obtained for 130 HER2-positive breast cancer patients do not support an influence of N-terminal brain natriuretic peptide (NT-proBNP), creatine kinase-MB (CK-MB), or myoglobin on the frequency of TIC. Suggestions for trastuzumab therapy include: close cooperation between cardiologists and oncologists; not using NT-proBNP, CK-MB, or myoglobin as standard TIC predictive markers; organizing prospective studies assessing the role of these parameters as TIC predictive markers in the case of HER2 blockage in conjunction with doublet immunotherapy or other anti-HER2 agents.

**Abstract:**

Trastuzumab-induced cardiotoxicity (TIC) can lead to early treatment discontinuation. The aim of this study was to evaluate: N-terminal brain natriuretic peptide (NT-proBNP), creatine kinase-MB (CK-MB), myoglobin, and selected biochemical and clinical factors as predictors of TIC. One hundred and thirty patients with HER2-positive BC receiving adjuvant trastuzumab therapy (TT) were enrolled. Measurement of cardiac markers and biochemical tests as well as echocardiography were performed prior to TT initiation and every three months thereafter. Cardiotoxicity leading to treatment interruption occurred in 24 patients (18.5%). While cardiotoxicity caused early treatment discontinuation in 14 patients (10.8%), the TIC resolved in 10 (7.7%) and TT was resumed. The most common complication was a decrease in left ventricular ejection fraction of more than 10% from baseline or below 50% (7.7%). In patients with TIC, there was no increase in the levels of NT-proBNP, myoglobin, and CK-MB. BMI, hypertension, ischemic heart disease, diabetes, age, cancer stage, type of surgery, use of radiotherapy, chemotherapy, and hormone therapy were shown to not have an effect on TIC occurrence. NT-proBNP, myoglobin, and CK-MB are not predictors of TIC. There is an ongoing need to identify biomarkers for TIC.

## 1. Introduction

Globally, breast cancer (BC) affects over one million women every year [1]. With the exception of Eastern Africa, BC is the most common cancer diagnosis all over the world, and a leading cause of cancer-related death in the female population of the majority of regions [2]. 

Currently, BC is characterized not only by clinical staging and pathological grading, but also by molecular hallmarks: cell proliferation marker Ki-67 and other markers, i.e., estrogen receptor (ER), progesterone receptor (PR), and human epidermal growth factor receptor 2 (HER2) [3]. Genomic analysis allowed identification of four molecular subtypes including: luminal A, luminal B, HER2-like, and basal-like (with predominant ER-, PR- and HER2-negative phenotype) [4]. New biomarkers are being identified for BC, for instance phosphatidylinositol-4,5-bisphosphate 3-kinase catalytic subunit alpha (PIK3CA) or programmed death-ligand 1 (PD-L1) [3].

HER2 expression is detected with the use of immunohistochemistry (IHC) and the amplification of the *HER2* gene with fluorescence in situ hybridization (FISH) [5]. HER2-positive and triple-negative tumors carry unfavorable prognoses; however, due to the introduction of anti-HER2 treatment, the overall survival (OS) and progression-free survival (PFS) within this group has improved significantly [4,6].

HER2-positive BC constitutes around 15–25% of all cases [4,7]. The treatment of locally advanced HER2-positive BC requires a combination of systemic therapy (chemotherapy, anti-HER2 agents +/− hormonal therapy), surgery and radiotherapy. Among these, anthracycline-based chemotherapy, monoclonal anti-HER2 antibodies (mAbs), and radiotherapy are considered to carry the risk of cardiotoxicity [7,8]. Anti-HER2 options for localized disease are: trastuzumab administered for one year, +/− pertuzumab in addition to trastuzumab as neoadjuvant treatment, and adjuvant antibody drug conjugate trastuzumab emtansine (T-DM1) in selected patients [7]. There are data supporting the idea of shorter trastuzumab treatment with non-inferior efficacy for 6 months vs 12 months and lower cardiotoxicity [9]. 

Numerous anticancer drugs are known to cause heart toxicity [10]. The risk of cardiovascular toxicity during the treatment of HER2-positive BC seems underestimated [11]. Proper imaging and biomarker assessment are important for prevention and early diagnosis as the efficacy of cardioprotective treatment remains questionable [11,12,13]. The data on cardiotoxicity incidence confirm the risk to be 3–26% for doxorubicin-based (type I cardiotoxicity) and 2–28% for trastuzumab treatment (TT) (type II)—trastuzumab-induced cardiotoxicity (TIC) [14]. Type I irreversible myocardial dysfunction is connected to myocardium destruction, while type II is related to the inhibition of physiological myocardial functioning, and is thought to be temporary [10,14,15]. However, numerous data show that TIC mechanisms are still evolving. HER2-positive BC patients commonly receive both types of systemic treatment: chemotherapy and antiHER2 treatment thus enhanced cardiotoxicity induced by anthracyclines and trastuzumab-based regimens can be noted [10,14,15].

According to data in the literature, the markers that can predict TIC in the BC HER2-positive population can be divided into [15,16,17,18,19,20]: biochemical: brain natriuretic peptide (BNP), N-terminal brain natriuretic peptide (NT-proBNP), troponins, myoglobin, creatine kinase-MB (CK-MB), glucose, uric acid, and lipids; morphological: decreased pre-treatment left ventricular ejection fraction (LVEF); clinical: history of hypertension, history of diabetes, radiotherapy treatment, older age, obesity (and overweight), race; genetic: *HER2* polymorphism.

The aim of our study is to determine the impact of selected cardiac biomarkers and other clinical and biochemical factors in predicting TIC in women treated radically for HER2-positive BC.

## 2. Materials and Methods

### 2.1. Study Population

The prospective study included 130 women with breast cancer treated radically at the Institute of Oncology in Krakow between 2009 and 2016. The research group was characterized by HER2 receptor overexpression and required the administration of trastuzumab as adjuvant setting. Women with HER2-positive BC who received TT adjuvant were eligible for study admission if (i) their baseline LVEF was >50% and (ii) no serious cardiological contraindications at baseline of study (1–2 weeks before the start of TT) were present.

The patients were treated according to standard guidelines that were valid during the treatment period (between 2009 and 2016) and following drug reimbursement in Poland. Therefore, neither neoadjuvant TT nor neoadjuvant chemotherapy was administered in this population. Primarily, the patients underwent radical surgical treatment followed by chemotherapy every 3 weeks with one of the schedules: (a) 4 cycles of doxorubicin 60 mg/m^2^ or epirubicin 60 mg/m^2^ plus cyclophosphamide 600 mg/m^2^ (AC/EC); (b) 4–6 cycles of doxorubicin or epirubicin 60 mg/m^2^ plus cyclophosphamide 600 mg/m^2^ followed by 1–4 cycles of docetaxel 75 mg/m^2^ (AC-T/EC-T); (c) 5-fluorouracil 500 mg/m^2^ plus doxorubicin or epirubicin 60 mg/m^2^ plus cyclophosphamide 500 mg/m^2^ (FAC). After chemotherapy, patients received radiotherapy if clinically indicated concomitantly with TT for a maximum of 1 year and adjuvant endocrine therapy in the case of a hormone-positive tumor for at least 5 years. Trastuzumab was administered intravenously 8 mg/kg as a loading dose followed by 6 mg/kg every 3 weeks. 

#### 2.1.1. Radiotherapy Technique

The RT technique depended on the type of surgical treatment. After breast-conserving surgery (BCS), the entire breast was irradiated applying the tangential fields technique, and when nodal involvement was presented, the mono-isocentric technique was used to irradiate the entire breast and regional lymph nodes. Additionally, all patients irradiated after BCS received a boost of 10 Gy to the tumor bed. In patients after mastectomy, the technique of monocentric mixed photon–electron beam was used. The target volume received a total dose of 45 Gy in 20 fractions given once a day, 5 days a week. 

#### 2.1.2. Primary Assessment (Baseline)

Before initiation of TT, the cardiovascular clinical risk factors of the patients had been assessed (body mass index [BMI], hypertension, presence of ischemic heart disease, diabetes, hyperlipidemia) [21] together with data concerning other comorbidities. The primary assessment was carried out by medical personnel working at the study site. Each patient was interviewed using the mandatory standard protocol at the study site and the information collected was included in medical records.

#### 2.1.3. Cardiac and Biomarker Evaluation

**Figure 1:**
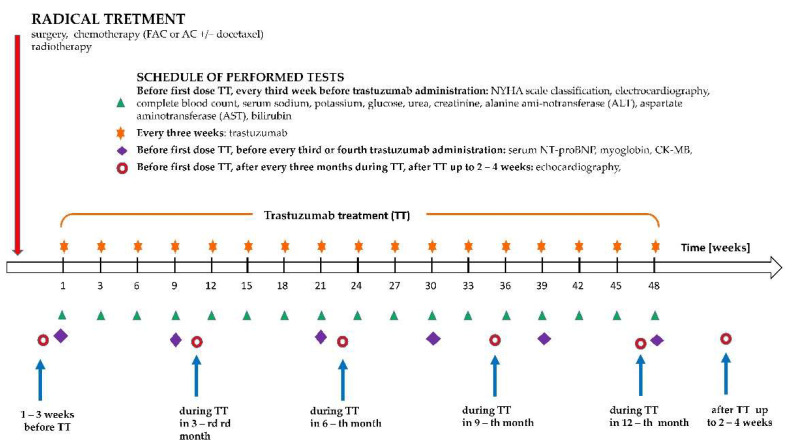
Study design schedule of performed measurements.

All patients underwent the following tests (Figure 1): (i) prior to every administration of trastuzumab (baseline) and then every three to four weeks—New York Heart Association (NYHA) scale classification, electrocardiography (ECG), complete blood count, serum sodium, potassium, glucose, urea, creatinine, alanine aminotransferase (ALT), aspartate aminotransferase (AST), and bilirubin; (ii) prior to the first administration of trastuzumab (baseline), then every three months—echocardiography (including evaluation of left ventricular ejection fraction [LVEF] and heart valve function), and (iii) before the first dose of trastuzumab (baseline), every three/four administrations of trastuzumab and before the last dose of trastuzumab: serum NT-proBNP, myoglobin, CK-MB, lipids, and uric acid.

If TIC was developed and fulfilled one or both of these values: (1) a reduction of LVEF of >10% (compared to the baseline value) or LVEF < 50% in asymptomatic patients; (2) symptoms manifestation of heart failure (according to the Framingham criteria) the treatment was interrupted [22]. These patients underwent re-evaluation in 2–3 weeks and, if their condition improved, TT was resumed. If not, it was stopped.

LVEF was evaluated using the biplane modified Simpson’s method in the apical two- and four-chamber views.

#### 2.1.4. Clinical Outcome

Data concerning oncological treatment for breast cancer (surgical treatment, chemotherapy, radiotherapy, hormone therapy) was collected by investigators from medical records. Echocardiography was performed by one experienced cardiologist. The blood sample was collected by medical stuff at the study site.

### 2.2. Laboratory Analysis

HER2 expression was assessed by immunohistochemistry (HercepTest). In the case of equivocal HER2 testing, *HER2* gene amplification was assessed by the FISH method. Blood for NT-proBNP, myoglobin, and CK-MB assessment was spun in a centrifuge (3800 × g/10 min). The obtained serum was stored at –70 Celsius degrees. The concentrations were measured applying the quantitative immunoenzymatic method using bioMérieux assays. All the samples were examined in the Analytics and Clinical Biochemistry Department at the Institute of Oncology, Kraków Branch. 

### 2.3. Ethical Considerations

Declaration: this study complies with the Declaration of Helsinki; the study received approval from the Ethical Committee at the Regional Medical Chamber in Kraków (decision from 4th December 2013, numbered B/NZ5/00764).

### 2.4. Statistical Analysis

Associations between categorical co-variates (comorbidities, BMI category, CK-MB, NT-proBNP, myoglobin, radiotherapy, hormone therapy, chemotherapy, valve insufficiencies, coexistent regurgitations) and the presence of cardiotoxicity (yes/no) during follow-up was statistically tested using Pearson’s Chi-squared test, or the Fisher’s exact test, for dichotomous co-variates. Repeated ANOVA measures were used to statistically test the change in LVEF level (%) over the follow-up, measured at six time points. Mann–Whitney U test assessed differences in mean value of 6 measurements for NT-pro-BNP, myoglobin, CK-MB (continuous variables) stratified according to age, BMI, presence of comorbidities, treatment, valvular in-sufficiency and LVEF. The results were considered as statistically valid if their p-value was equal to or below 0.05.

The correlation coefficient (r) was calculated to assess decrease and increase of LVEF value during trastuzumab therapy. We indicated 4 grades of LVEF decrease or increase: (1) strong decrease (r ≤ −0.6), (2) weak decrease (r > −0.6 and r ≤ 0), (3) weak increase (r > 0 and r ≤ 0.6), (4) strong increase (r > 0.6).

## 3. Results

### 3.1. Baseline characteristics

One hundred and thirty women with HER2-positive BC (aged 33–77 at the onset of TT) were enrolled in the study. In the whole group, all tests were performed using the required protocol.

Table 1 presents the baseline characteristics and the applied treatment methods in the study participants. All patients underwent radical surgical treatment (Madden radical mastectomy or breast-conserving surgery), anthracycline-based adjuvant chemotherapy (AC/EC, AC-T/EC-T, FAC) and—in the case of the hormone receptor-positive breast cancer—received hormonal therapy. Postoperative radiotherapy (RT) was delivered in 102 patients (78.5%) using a linear accelerator (Table 1). Overall, the patients received 1–19 cycles of TT.

### 3.2. Key Outcome Measures

During TT, cardiac complications were detected in 24/130 (18.5%) patients (Table 2). Of the 24 patients with cardiac complications: 14 (10.8% of the total number of patients) developed side-effects which, due to their persistence, resulted in early treatment discontinuation; in 10 (7.7%), therapy was resumed and applied in accordance with the protocol after temporary TT suspension and side-effects improvement.

#### 3.2.1. Cardiac Complications Prevalence, Clinical Parameters, and Risk Factors

Analysis of changes in prevalence of cardiac complications through trastuzumab treatment (using repeated-measure ANOVA) revealed that variables such as: age, BMI, presence of hypertension, ischemic heart disease, diabetes mellitus, stage of the breast cancer, type of surgical treatment, application of radiotherapy, hormone therapy, or chemotherapy may not affect the occurrence or change in the severity of complications during treatment with trastuzumab (data not presented). 

**Figure 2:**
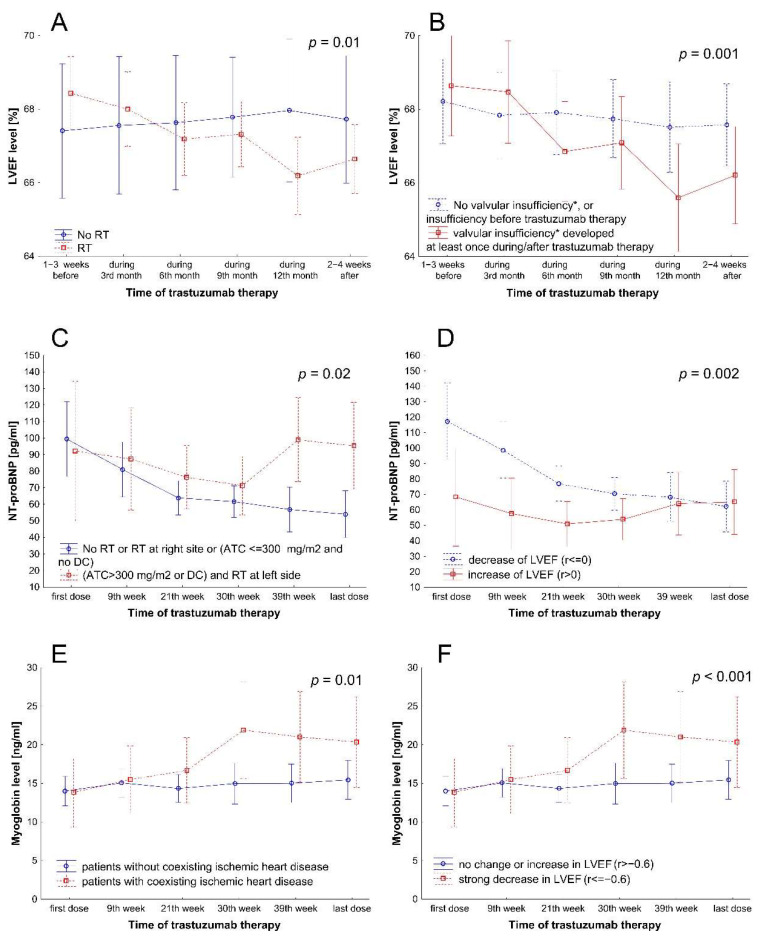
Changes during therapy in levels of LVEF in patients with or without radiotherapy (**A**) and in patients with different occurrence of valvular insufficiency of any grade (**B**); in levels of NT-proBNP in patients stratified according to treatment (**C**) or changes in LVEF (**D**); in levels of myoglobin in patients with or without coexisting ischemic heart disease (**E**) and in patients stratified according to LVEF changes (**F**). * Valvular insufficiency of any grade. Abbreviations: ATC, doxorubicin (anthracycline); DC, docetaxel; LVEF, left ventricular ejection fraction; NT-proBNP, N-terminal brain natriuretic peptide; RT, radiotherapy.

The decrease of LVEF value was most significant in patients who had received radiotherapy (*p* = 0.01, Figure 2A) and those who developed valve insufficiency during or after radiotherapy (but not before) TT (*p* = 0.001, Figure 2B). Other studied parameters were not related to changes in LVEF values before/during/after targeted therapy.

#### 3.2.2. TIC Occurrence According to Levels of Clinical and Biochemical Parameters 

Table 3 presents the relationship between comorbidities, BMI category, CK-MB, NT-proBNP, myoglobin, radiotherapy, hormone therapy, chemotherapy, valve insufficiencies, coexistent regurgitations) and the presence of cardiotoxicity. Univariate analyses showed that none of the clinical and biochemical parameters (including NT-proBNP, myoglobin, CK-MB and their changes during therapy) were significantly associated with risk of TIC occurrence. Therefore, we conclude that the usefulness of NT-proBNP, myoglobin, CK-MB as TIC markers is limited.

The NT-proBNP level (the mean value of 6 measurements before/during/after TT) was significantly higher in: older patients; patients who received a more aggressive chemotherapy schedule (anthracyclines > 300 mg/m^2^ or docetaxel) and simultaneously RT at the left site; women who developed valvular insufficiency of any grade during or after (not before) TT and those with a decrease in LVEF during TT (*p* < 0.05, Table 4).

Moreover, with repeated-measure ANOVA, we found a significant increase in the level of NT-proBNP in patients who had received a more aggressive chemotherapy schedule and RT at left site (*p* = 0.02, Figure 2C). At the same time in patients with less aggressive therapy (anthracyclines≤300 mg/m^2^ and no docetaxel) or lack of RT or RT at right side), the NT-proBNP level decreased (*p* = 0.02, Figure 2C). Moreover, the decrease in NT-proBNP values was more pronounced in patients who had developed a decrease of LVEF values during TT (*p* = 0.002, Figure 2D).

The myoglobin level (the mean value of 6 measurements before/during/after TT) was significantly higher in older patients (*p* = 0.02, Table 4). Moreover, with repeated-measure ANOVA, we found a significant increase in the myoglobin level in women with a coexisting ischemic heart disease and those whose LVEF significantly decreased (r ≤ −0.6) before/during/after TT (*p* = 0.01, *p* < 0.000 respectively, Figure 2E,F).

Finally, we analyzed the relationship between risk factors (age, BMI, coexisting diabetes mellitus, arterial hypertension, and ischemic heart disease) and the level of biochemistry tests (the mean value of 6 measurements before/during/after TT). Moreover (as described above), a significant increase in myoglobin level was found in women with a coexisting ischemic heart disease, while in those without this condition, the level of this marker decreased.

The serum NT-proBNP level (counted as a mean value from six measurements before, during and after TT) was significantly increased among: older patients (>54 years); patients who received both left-sided RTH and more aggressive chemotherapy (anthracyclines >300mg/m^2^ or docetaxel), but it did not correlate with the TIC occurrence.

Multivariate analysis was not conducted since there were only 24 “events” (TICs). A general rule developed by F. Harrell was used, which states that to have an adequate statistical power, multivariate analysis should include at least 10 events per variable. This results in a maximum of only 2–3 variables that can be safely included in multivariate analysis for our data.

## 4. Discussion

HER2 is a transmembrane receptor with tyrosine kinase activity. It is involved in a transduction of signals that induce cell growth and differentiation. HER2 overexpression or amplification is a negative prognostic factor for patients with BC and is predictive of response to anti-HER2 treatment. Anti-HER2 therapies significantly improve the prognosis of HER2-positive BC patients by blocking HER2 signaling. However, HER2 also plays an important role in maintaining a proper function of cardiomyocytes [23]. HER2 exhibits a high affinity for dimerization with the entire family of epidermal growth factor receptors (EGFR), especially with HER4 in cardiomyocytes, which is crucial for heart functioning. Neuregulin, a protein binding to and activating both HER2 and HER4 tyrosine kinases, supports growth, organization of myofilaments, and survival of isolated cardiomyocytes. It also protects cardiomyocytes from stress and reactive oxygen species, induces cell division cycle, and promotes regeneration, improving heart function. A disruption of HER2 signaling antagonizes neuregulin’s effects [24].

Recently, there has been a growing interest in TIC. As for now, neither its risk factors nor predictive factors have been clearly identified. In our study, TIC occurred in 18% of patients, leading to a premature termination of therapy in approximately 10% of these cases. TIC was developed more often than in trastuzumab registration studies. Their authors observed an incidence of up to 4.1% of cardiotoxicity in patients assigned to the trastuzumab-containing arm in the NASBP-31 trial [25,26,27,28]. However, in the retrospective cohort study by Bowles et al., an asymptomatic TIC was diagnosed in 9.8% of 12,500 BC patients, suggesting that TIC occurs more often in clinical practice than had been supposed [29]. This hypothesis is supported by a retrospective analysis of 450 BC patients by Sato et al., with TIC occurrence of 9.2%. Moreover, they observed that factors commonly thought to be predictors for cardiac toxicity for TT: age, BMI, hypertension, ischemic heart disease, diabetes mellitus and treatment with anthracyclines, are not TIC predictors. Only the development of valvular heart disease was proved to be a TIC predictive factor [30,31]. Similarly, our analysis shows that age, BMI, hypertension, ischemic heart disease, diabetes mellitus, radiotherapy, and treatment with anthracyclines (with or without docetaxel) are not TIC predictive factors. These data stand in opposition to the opinion of the European Society of Cardiology, which states that age, BMI >30kg/m^2^, hypertension, heart disease, anthracyclines therapy, and radiotherapy increase the risk of TIC [32]. This proves that we do not have clinical predictors of TIC, and the commonly used method of TIC diagnosis based on echocardiography and LVEF assessment is not optimal. There are studies proving that the application of new techniques such as cardiac magnetic resonance imaging (MRI), positron emission tomography (PET)/MRI and myocardial scintigraphy may facilitate TIC recognition [33]. The problem lies in their high cost and low availability. Still, echocardiography is the least expensive and most available diagnostic tool. Therefore, it is crucial while performing echocardiography to assess heart valves and left ventricular relaxation impairment since it may precede systolic dysfunction [34]. Whether cardiac markers might be used as TIC predictors is still unclear [35,36]. This study shows that a higher incidence of TIC does not correlate with an elevated level of the following markers: NT-proBNP, CK-MB, and myoglobin measured before TT. In addition, no rise in these parameters was observed among patients who developed TIC during the immunotherapy. The analysis of the patients who had to stop the treatment due to cardiac complications demonstrates that there were no changes in the NT-proBNP level just before the occurrence of the adverse effects. This leads to the conclusion that changes in the NT-proBNP level cannot be used as a predictive factor of TIC. A review of literature is inconclusive in terms of the usefulness of NT-proBNP measurement in predicting cardiac complications during oncological treatment [13,36,37,38]. Although Meinardi et al. noted a rise in the NT-proBNP level during a year-long observation of 40 patients undergoing adjuvant anthracycline-based therapy, this finding did not correlate with left ventricular systolic dysfunction [39]. On the other hand, an elevation of NT-proBNP level was associated with impairment of left ventricular systolic and relaxation function among 26 patients with acute leukaemia treated with anthracyclines [38]. Moreover, Sandri et al. showed that a maintained increased NT-proBNP level over 72 h after the end of therapy correlates with development of left ventricular systolic and relaxation dysfunction during a year-long observation [37]. Similarly, the usefulness of other markers, such as myoglobin or CK-MB, has not been confirmed. Horacek et al. did not observe significant changes in their level in patients with acute leukemia treated with anthracyclines [40]. Ambiguous results of the aforementioned studies, differing methods of measurement of these markers and assessment of heart function as well as heterogeneity of analyzed groups do not permit the formulation of clear conclusions and recommendations for measurement of NT-proBNP and other cardiac markers in early diagnosis of TIC among BC patients. Interestingly, TIC has been proved to occur more often among patients of Afro-American origin, suggesting that genetic factors might be predictive of TIC. According to Stanton et al. *HER2* single nucleotide polymorphism (Pro 1170 Ala) is associated with a 2.6 × higher risk of TIC [41].

### Study Limitations

This research has certain limitations. It is a single center study with established cooperation between oncologists and cardiologists, which might not reflect all oncology sites conducting such treatment in HER2-positive BC population. Almost all patients were pretreated with anthracyclines, thus the conclusions can be drawn for this population.

In this research we did not obtain data for troponin levels. Troponin I was suggested at the time of our study initiation as a biomarker of TIC [36]. Thus, we aimed to search for new predictors. However, numerous data are inconclusive with current results obtained for clinical trial patients showing no significance in terms of TIC for Troponin T [42]. This topic requires further evaluation.

The current treatment of BC has moved from the adjuvant to neoadjuvant setting and from single anti-HER2 blockage to double blockage with pertuzumab and adjuvant T-DM1 in selected populations [43]. For this reason, conducting a trial or even an observational study with a larger population that assesses the potential toxicity of single HER2 blockage with trastuzumab in a BC population and incidence of TIC would be challenging. Thus, it is necessary to rely on data obtained before the change of practice (prior to 2016). In this context, this study’s data, collected prospectively on a population of 130 women, are unique.

Another limitation is the small group of patients with cardiac complications, which were detected during trastuzumab treatment. In this study, complications were observed in 24 patients (18.5%). In such a small group, random events could significantly affect the results.

## 5. Conclusions

A comprehensive oncological BC treatment is associated with the risk of cardiovascular complications. It is necessary to properly qualify patients for treatment and cardio-monitoring during treatment, in accordance with the Recommendations of the National Team for Cardiology and Oncology Supervision [44]. Cardio-monitoring facilitates the early diagnosis of heart failure and the resulting implementation of cardiological treatment. In many cases, it is necessary to modify or discontinue oncological treatment. The observation carried out in this study showed that, in addition to symptomatic heart failure and a decrease in LVEF, there are a number of other cardiovascular disorders associated with oncological treatment [31]. It seems that the frequency and severity of cardiac complications are underestimated in the clinical trial population and seem to be higher in the real-world data. There is no evidence for the usefulness of cardiological biomarkers such as: NT-proBNP, CK-MB, or myoglobin in predicting cardiological complications. The challenge is the effective treatment of BC with minimal toxicity. This is especially important when using adjuvant therapy. In these patients, late effects of oncological treatment may develop after several decades and become a serious health issue. Taking into account the existing and constantly evolving knowledge of the types and mechanisms of cardiotoxicity as well as the methods for its prediction, detection and monitoring, it seems that cardio-oncological care should be more comprehensive in order to minimize the toxicity of BC treatment [45,46,47].

### Clinical Implications

Suggestions for BC patients receiving TT include close cooperation between cardiologists and oncologists; inclusion of a cardiologist as a Breast Unit team member; not using NT-proBNP, CK-MB, or myoglobin as standard TIC predictive markers; also including patients without cardiac risk factors in close cardiac monitoring; in patients with TIC, a close follow-up of cardiac toxicity is recommended as TT can be resumed in the majority of patients.

## Figures and Tables

**Table 1:** Patients’ baseline characteristics.

Parameter		No. of Patients (%)
Surgical treatment	BCT	99 (76.2)
	Madden radical mastectomy	31 (23.8)
Stage of breast cancer	I	32 (24.6)
	IIA	52 (40.0)
	IIB	22 (16.9)
	IIIA	24 (18.5)
Laterality	Left	67 (51.5)
	Right	63 (48.5)
Treatment	Radiotherapy	102 (78.5)
	Hormonal therapy	66 (50.8)
	Chemotherapy	127 (97.7)
	Docetaxel administration	34 (26.2)
Anthracycline dose [mg/m^2^]	0	2 (1.5)
	240	73 (56.2)
	300	4 (3.1)
	360	49 (37.7)
	480	2 (1.5)
Comorbidities	Ischemic Heart Disease	19 (14.6)
	Hypertension	34 (26.2)
	Type II Diabetes	5 (3.8)
BMI [kg/m^2^]	19–25	40 (30.8)
	25–30	49 (37.7)
	>30	41 (31.5)

Abbreviations: BCT, breast conserving therapy; BMI, body mass index.

**Table 2:** Cardiac complications during trastuzumab therapy.

Complication	All Cases: 130		
	No. of Cases with Interruption of TT	No. of Cases with Cessation of TT	No. of TT Cycles in Patients with TT Cessation
Decrease in LVEF	10 (7.7%)	3 (2.3%)	7, 8, 12
Decrease in LVEF + heart failure	2 (1.5%)	2 (1.5%)	1, 11
Decrease in LVEF + arrhythmia	1 (0.8%)	0	-
Decrease in LVEF + arrhythmia + heart failure	1 (0.8%)	1 (0.8%)	6
Heart failure	3 (2.3%)	3 (2.3%)	7, 15, 15
Arrhythmia	1 (0.8%)	0	-
Arrhythmia + heart failure	1 (0.8%)	0	-
Severe valvular regurgitation	2 (1.5%)	2 (1.5%)	9, 12
Cardiac conduction disorder	1 (0.8%)	1 (0.8%)	11
Exacerbation of coronary artery disease	2 (1.5%)	2 (1.5%)	4, 12
Total	24 (18.5%)	14 (10.8%)	-

Abbreviation: LVEF, left ventricular ejection fraction.

**Table 3:** Relation between clinical and biochemical parameters and TIC.

Parameter		No. of Patients	Cardiotoxicity Present in *n* (%)	*p*
Ischemic Heart Disease	Yes	19	4 (21.1)	0.75 ^a^
	No	111	20 (18.0)	
Hypertension	Yes	34	7 (20.6)	0.80 ^a^
	No	96	17 (17.7)	
Diabetes mellitus	Yes	5	1 (20.0)	1 ^a^
	No	125	23 (18.4)	
BMI	Normal range	40	6 (15.0)	0.19 ^b^
	Overweight	49	13 (26.5)	
	Obese	41	5 (12.2)	
NT-proBNP	Elevated	12	3 (25.0)	0.696 ^a^
	Within normal limits	117	21 (17.9)	
Myoglobin	Elevated	46	8 (17.4)	1 ^a^
	Within normal limits	83	16 (19.3)	
CK-MB	Elevated	4	1 (25.0)	0.57 ^a^
	Within normal limits	125	23 (18.4)	
Increase in NT-proBNP level during therapy ^c^	Yes	10	2 (20.0)	1 ^a^
	No	118	21 (17.8)	
Increase in myoglobin level during therapy ^c^	Yes	57	10 (17.5)	1 ^a^
	No	71	13 (18.3)	
Increase in CK-MB level during therapy ^c^	Yes	9	3 (33.3)	0.20 ^a^
	No	119	20 (16.8)	
Radiotherapy	Yes	102	19 (18.6)	1 ^a^
	No	28	5 (17.9)	
Hormone therapy	Yes	66	12 (18.2)	1 ^a^
	No	64	12 (18.7)	
Chemotherapy	Yes	127	24 (18.9)	1 ^a^
	No	3	0 (0.0)	
Docetaxel	Yes	34	9 (26.5)	0.21 ^a^
	No	93	15 (16.1)	
No. of docetaxel cycles	4	23	4 (17.4)	0.08 ^b^
	1–3	11	5 (45.4)	
	0	93	15 (16.1)	
Anthracycline dose [mg/m²]	240 or 300	77	13 (16.9)	0.64 ^a^
	360 or 480	51	11 (21.6)	
Mitral valve insufficiency	Yes	90	18 (20.0)	0.45 ^a^
	No	39	5 (12.8)	
Tricuspid valve insufficiency	Yes	30	5 (16. 7)	1 ^a^
	No	99	18 (18.2)	
Aortic valve insufficiency	Yes	24	5 (20.8)	0.77 ^a^
	No	105	18 (17.1)	
Coexistent regurgitations	Yes	98	20 (20.4)	0.28 ^a^
	No	31	3 (9.7)	

^a^ Fisher exact test, ^b^ Pearson Chi^2^, ^c^ value higher than baseline in at least one of measurements during/after TT. Abbreviations: BCT, breast conserving therapy; BMI, body mass index. The following normal range values were applied: NT-proBNP < 250 pg/mL; myoglobin 10–46 ng/mL; CK-MB < 5.1 ng/mL; Attention: Among a few patients only one measurement of NT-proBNP/myoglobin/CK-MB level was conducted. These patients were not taken into consideration during the analysis.

**Table 4:** Relationship between chosen clinical parameters and cardiac complications/valvular regurgitation developed during trastuzumab therapy.

			Mean Value of 6 Measurements before/during/after Trastuzumab Therapy		
Parameter	Category	N	NT-pro-BNP Mean (SD)	M Mean (SD)	CK-MB Mean (SD)
	Total	129	88.4 (116.8)	16.8 (20.9)	3.0 (9.2)
Age	≤54 years	66	70.9 (102.8)	13.7 (7.7)	2.0 (0.9)
	>54 years	63	106.8 (128.2) ^a^	20.1 (28.6) ^b^	4.0 (13.1)
BMI	Normal weight: BMI ≤ 25	48	96.8 (122.1)	14.1 (8.2)	1.9 (0.8)
	Overweight: BMI > 25	81	83.5 (114.1)	18.5 (25.6)	3.6 (11.5)
DM	Not present	124	89.4 (118.8)	17.0 (21.3)	3.0 (9.4)
	Present	5	65.3 (43.3)	12.8 (6.6)	1.8 (1.0)
AH	Not present	96	88.2 (131.1)	17.2 (23.8)	3.3 (10,6)
	Present	33	89.0 (59.5)	15.8 (8.1)	2.0 (1.2)
IHD	Not present	110	85.9 (123.2)	16.7 (22.23)	3.1 (9.9)
	Present	19	103.2 (70.2)	17.4 (10.98)	2.0 (0.9)
RT or ChT	No RT or RT at right site or (ATC ≤ 300 mg/m^2^ and no DC)	97	77.6 (92.5)	14.5 (8.8)	2.2 (2.1)
	(ATC > 300 mg/m^2^ or DC) and RT at left side	32	121.4 (168.4) ^b^	23.8 (38.8)	5.3 (18.1)
HT	Not administered	62	103.2 (157.0)	19.4 (28.7)	4.0 (13.2)
	Administered	67	74.8 (57.3)	14.4 (8.6)	2.0 (1.0)
Valvular insufficiency of any grade *	Not present or present before and during/after trastuzumab therapy	67	65.8 (52.1)	15.8 (10.1)	2.4 (2.4)
	Developed during or after (not before) trastuzumab therapy	47	79.1 (48.4) ^b^	14.3 (8.0)	2.0 (1.1)
LVEF	Decrease during/after trastuzumab therapy *	79	103.6 (141.1)	17.8 (26.2)	3.6 (11.7)
	Increase during/after trastuzumab therapy **	48	61.8 (50.0) ^a^	15.6 (7.0)	2.0 (0.8)

Mann-Whitney U test: ^a^, *p* = 0.001; ^b^, *p* = 0.02; statistically significant relationship were marked as bold. Abbreviations: BMI, Body Mass Index; ATC, anthracyclines; DC, docetaxel; RT; radiotherapy; ChT, chemotherapy; HT, hormonotherapy, DM, diabetes type 2; AH, arterial hypertension; IHD, Ischemic Heart Disease; NT-pro-BNP, [pg/mL]; M, myoglobin [ng/mL]; CK-MB mass [ng/mL]; *: r ≤ 0, **: r > 0.

## Data Availability

Data available on request.
